# “I wouldn’t have hit you, but you would have killed your baby:” exploring midwives’ perspectives on disrespect and abusive Care in Ghana

**DOI:** 10.1186/s12884-019-2691-y

**Published:** 2020-01-06

**Authors:** Veronica Millicent Dzomeku, Adwoa Bemah Boamah Mensah, Emmanuel Kweku Nakua, Pascal Agbadi, Jody R. Lori, Peter Donkor

**Affiliations:** 1 Department of Nursing, Faculty of Allied Health Sciences, College of Health Sciences, Kwame Nkrumah University of Science and Technology, Kumasi, Ghana; 2 Department of Epidemiology and Biostatistics, School of Public Health, Kwame Nkrumah University of Science and Technology, Kumasi, Ghana; 3 Department of Health Behavior and Biological Sciences, School of Nursing, University of Michigan, Ann Arbor, Michigan USA; 4 Department of Surgery, School of Medical Sciences, Kwame Nkrumah University of Science and Technology, Kumasi, Ghana; 5 Komfo Anokye Teaching Hospital, Kumasi, Ghana

**Keywords:** Disrespectful maternity care, Childbearing women, Midwives, Ghana, Qualitative

## Abstract

**Background:**

Quality maternal health reduces maternal and neonatal mortality and morbidity. Healthcare professionals, including midwives, are significant agents for the promotion of quality maternal health. Frequents reports of disrespect and abuse of childbearing women by midwives during intrapartum care are becoming common, suggesting that many of these agents are engaging in care practices that compromise quality maternal health. Thus, understanding midwives’ descriptions and experiences of the phenomenon is critical to addressing the threat. This paper, therefore, explored the understanding of midwives on D&AC and their occurrence in professional practice in a tertiary health facility in Kumasi, Ghana.

**Methods:**

An exploratory descriptive qualitative research design using an interpretative approach was employed in the study. Data were generated through individual in-depth interviews. Data saturation was reached with fifteen interviews. The interviews were audio-recorded and transcribed verbatim. Open Code 4.03 was used to manage and analyse the data.

**Results:**

The midwives understood D&AC. They also confirmed meting out or witnessing colleagues engage in D&AC in their professional practice. The midwives described D&AC as the provision of inadequate care and the overlooking of patient-centred care, and verbal, physical, and psychological abuse. The themes revealed that socio-economic inequalities, provider perception and victim-blaming, and health system-related factors facilitate D&AC. It emerged that the following marginalized groups were at high risk for D&AC: the non-compliant, mentally ill, HIV/AIDs+, teenagers, poor, and childbearing women on admission at the general labour ward.

**Conclusion:**

The midwives understood D&AC and revealed that it frequently occurred in their professional practice. Frequent in-service training on respectful maternity care and monitoring of care provision in healthcare facilities are needed to eliminate the incidence of D&AC.

## Background

Global interventions to reduce maternal deaths have yielded notable, positive results [1]. The global maternal mortality estimate reduced from 532,000 in 1990 to 303,000 in 2015, representing a decrease of approximately 44% [1]. About 66% of this current estimate occurred in sub-Saharan Africa, suggesting marginal achievements in this region [1]. Ghana’s maternal mortality ratio is 319 per 100,000 live births, which represents about 50% reduction from the 1990 estimate [2]. This figure, however, falls short of the 75% Millennium Development Goal (MDG) target [2]. Therefore, there is a continued need to identify and examine solutions in sub-Saharan Africa in targeted scientific research, policy design, programs, and interventions at continental, national, and community levels [3, 4].

Facility-based delivery with skilled providers is an essential solution that has attracted research and policy interests over time because of its promise of drastically reducing maternal deaths in sub-Saharan Africa [5, 6]. Thus, many countries have invested in training skilled birth attendants and creating maternity units in healthcare facilities [7–9]. With enough investments and campaigns, the continent has witnessed a rise in the number of women who choose to deliver in healthcare facilities [10, 11]. For instance, Ghana’s facility-based delivery with a skilled provider increased from an estimated 40% in 1988 to 74% in 2014 [12].

Like many countries in low and middle-income countries (LMICs), the reduction in maternal deaths in Ghana has been attributed to the continuous rise in facility-based deliveries [13]. This success, although slow, is likely to stagnate or even decline due to frequent reports of disrespect and abusive maternal care in many healthcare facilities. Home delivery, an alternative to facility-based care, have its own negative maternal and neonatal outcomes. Homes generally lack emergency equipment and often without skilled providers, so delivery complications often result in preventable disabilities, morbidities, and maternal and neonatal deaths [14, 15]. In Ghana, skilled-birth delivery occurs only in healthcare facilities.

Disrespect and abusive care (D&AC) is defined as the “interactions or facility conditions that local consensus deem to be humiliating or undignified, and those interactions or conditions that are experienced as or intended to be humiliating or undignified” [16]**.** Scholarly classification of facility-based D&AC encapsulates these key domains: physical abuse, non-consented care, non-evidence-based care, non-dignified care, non-confidential care, abandonment, discrimination, and detention [17, 18]. In consonance with this, studies have documented the forms of D&AC childbearing women have experienced in healthcare facilities. The women in these studies reported that they were physically abused [19], psychologically abused [19], detained for non-payment of bills [20], examined without consent [21, 22], discriminated against because of their social status [21, 22], denied of their choice of birthing position because of facility-recommended guidelines [22–24], and subjected to iatrogenic procedures such as episiotomies, in some instances done without anaesthesia, and with improper pelvic examinations [25]. This D&AC, Sen, Reddy & Iyer [26] argued, is driven by socioeconomic inequalities and institutional structures and processes.

The impact of D&AC on maternal and neonatal death could be both direct and indirect. Evidence suggests that subjecting childbearing women to any form of abuse during labour and delivery may result in the death of either mother or baby or both [13]. Indirectly, D&AC in healthcare facilities have compelled childbearing women to deliver their babies at home, which predisposes both mother and baby to conditions that threaten their survival [14, 15]. The threat D&AC poses to the health and lives of women and children violates their basic human rights to life, dignity, and quality of life [27, 28].

Studies from countries other than Africa exploring maternity care providers’ views on D&AC have reported interesting findings [23]. From a meta-ethnographic study, it is evident that midwives in the United Kingdom (UK) were aware of childbearing women’s rights to autonomy but were often faced with a dilemma when childbearing women make birth position choice outside recommended guidelines [23]. Additional cited reasons for the compromise of quality and respectful intrapartum care as reported by Indian providers were non-cooperative attitudes of childbearing women and their family as well as structural layout of labour units which does not ensure privacy in labour [29].

Studies on midwives’ experiences of disrespect and abuse of childbearing women during intrapartum care seem limited in sub-Saharan Africa [30–33]. The few evidence on the phenomenon have reported frequent cases of disrespect and abusive care during childbearing women’s labour and delivery, with weak health systems and intent to save mother and baby from death commonly cited as reasons for engaging in D&AC practices [30, 31, 34, 35]. For instance, a Ghanaian study involving student midwives revealed that although they understood what constituted D&AC, these student midwives mentioned that some forms of D&AC were justified when the intent was to save both mother and baby from dying during delivery [34].

Over the past decade, most studies on D&AC have explored the views of postpartum women in Ghana on the phenomenon [19, 36, 37]. In reporting that D&AC is commonplace in many healthcare settings in Ghana, postpartum women expressed their dissatisfaction with such practices and some of these women resorted to either avoid facilities with D&AC reputations or deliver their next child at home [19, 36]. To complement postpartum women views on the phenomenon, the present study seeks to add to this growing body of knowledge by answering the following research question: What are the descriptions of D&AC by midwives and their occurrences in their professional practice? Answers to this question are necessary because midwives are at the frontline of maternity care and their understanding of D&AC has policy implications for quality maternity care, curriculum restructuring, and in-service training for skilled birth attendants.

### Design

An explorative, descriptive qualitative design using an interpretative approach was employed to explore the experiences and views of midwives on disrespectful and abusive maternal care in their professional practice. This design was chosen because it permits the authors to explore and document midwives’ understanding, experiences of, and their interpretations of actions that are deemed D&AC [38]. The COREQ checklist for reporting qualitative research guided the study design and write-up [39].

### Study setting

The study setting was in a tertiary health facility in Kumasi, located in the Ashanti region of Ghana. It serves patients across the country and has a bed capacity of about 1200 and a staff strength of about 3000. It is the main referral hospital for the Ashanti, Brong Ahafo, Western, the three northern regions (Northern, Upper East, And Upper West), and neighbouring countries. It has twelve (12) directorates (departments) one of which is the Obstetrics and Gynaecology (O &G) directorate, which has four labour wards. In 2018, the hospital recorded an estimated 4792 Spontaneous vaginal deliveries, an estimated 123 maternal deaths, and 61 neonatal deaths [KATH O & G Records, 2019].

### Population, sampling, and sample size

The study population constituted midwives in a tertiary health facility in Kumasi, Ghana. The inclusion criteria for the study were midwives who have had at least one year of professional practice and were working on the labour ward. We sampled participants purposively from a total number of 96 midwives who were currently working in the labour wards of KATH. The second author approached these midwives individually, discussed the study and obtained written consent prior to the interviews. Four interviews were conducted every week to allow for transcription and coding to ascertain patterns of emerging themes. The interviews ended with the 15th participant, as no new information or theme emerged [40, 41].

### Data collection

Data were collected through face-to-face in-depth interviews using a semi-structured interview guide which was designed based on a respectful maternal care module (RMC-M) developed by the first author in her preliminary studies [42]. The guiding questions were also informed by the study’s objectives and existing literature on respectful and abusive maternal care and reviewed by experts in maternal care. The guide included probing questions that ensured an exhaustive exploration of participants view and account of the phenomenon. Example of the guiding question were; “Please, in your opinion, what is respectful care?” “Please, in your view, what is non-abusive care?” The interview guide has been attached as a supplementary file [Additional file 1]. Data collection started on 3rd January 2019 and ended on 25th February 2019. The interview guide was pre-tested with 3 midwives working at the labour ward on the maternal unit of the Kwame Nkrumah University of Science and Technology Hospital, Kumasi to ensure the appropriateness of the guiding questions. The interviews were conducted by the second author (ABBM), a qualitative researcher with clinical and academic experience in women’s health and maternal care. As a researcher, ABBM speaks and writes both ‘Twi’ and English languages. The interviewer does not work at KATH; hence she had no direct influence on the study setting and the participants. Additionally, ABBM is a female and has several years of experience as a nurse; therefore, she knew which questions to ask and could identify with the participants, which heightened the validity of the study. The interviews language was English. The estimated interview duration was about 50 to 80 min, and the interviews were audio-recorded with the participants’ consent. Venue (office at KATH), date, and time of the interviews were scheduled to suit the participants. Field notes were taken during each interview to include non-verbal cues and other relevant observations during the interview process.

### Data management and analyses

Data were analysed concurrently with data collection using a thematic analytical approach. All the interviews were transcribed verbatim by the first and second authors. The first author (VMD) is a qualitative researcher with clinical and academic experience in women’s health, maternal care and midwifery education. VMD speaks and writes both ‘Twi’ and English languages and does not work at KATH. Prior to a verbatim transcription of the audio-recorded interviews, the researchers thoroughly listened to the audio files. The transcribed interviews were independently proofread by the third and fourth authors (with broad academic and research backgrounds in public health and biostatistics) to ensure the participant’s views were precisely captured. Anonymity was ensured by serializing each transcript file, and the transcripts were kept in a secured folder on the laptop of the principal investigator. Open Code 4.03, a qualitative data management software, was used to manage the data for analysis. The first and second author analysed the data independently and this was independently confirmed by the fourth author and validated by the third author. The D&AC project was created in the software, and the transcripts were saved as text files and imported into the project folder. Each transcript was coded, and the codes synthesized into subthemes and further into themes based on their similarities and relationships [43, 44]. The themes that emerged structured the presentation of the findings.

### Trustworthiness/rigour

Trustworthiness was ensured using the following criteria: confirmability, transferability, dependability and authenticity [45]. Employing purposive sampling techniques ensured that participants who had the relevant experience on the subject of study were enrolled in the study. Confirmability was achieved through member checking with four participants and this ensured that participants’ realities were accurately presented before drawing the final conclusions of the data [45]. Also, independent analysis and validation of the data by the authors further confirmed the findings. A detailed description of the study methodologies, design, and setting, as well as the background of the participants, ensured transferability and the potential replication of the study by future researchers. Through peer debriefing and strict adherence to the study protocol, the trustworthiness of the data was further ensured.

## Results

### Demographic features of participants

The midwives were on average 33 years old, with a range of 31–48 years. They had engaged in professional practice for an average of eight years. Seven participants obtained a bachelor’s degree in midwifery and the remaining a diploma. Only one of the midwives was a Muslim, and the others were Christians. Eleven were currently married. Those with children (*n* = 10) had an average of 2.3 living children (range = 1–3).

Views of the midwives were sought on disrespect and abusive care (D&AC) and the occurrence in their professional practice. Three main themes emerged from the data: (1) Inadequate intrapartum care and forms, (2) facilitators of D&AC, and (3) everyday occurrences of D&AC. The themes also had sub-themes as presented in Table 1. The codes associated with the themes and subthemes are reported in a supplementary file [Additional file 2].

**Table 1:** Themes and Subthemes

Themes	Sub-themes
Inadequate intrapartum care and forms	1. Providing inadequate care and overlooking patient-centred care
	2. Forms of Abuse (verbal, psychological, and physical)
Facilitators of D&AC	1. Discriminatory care
	2. Provider perception and victim-blaming childbearing women/
	3. Non-evidenced based practices of preventing adverse outcome
	4. Health systems problems
Everyday occurrences	1. Everyday occurrences

### Inadequate intrapartum care and forms

All the midwives were aware of D&AC, and their descriptions of D&AC are categorized as providing inadequate care & violation of patient-centred care, and forms of abuse (verbal, psychological, and physical). Also, their views on the prevalence of D&AC are presented.

#### Providing inadequate care & violation of patient-centred care

The midwives believed that providing suboptimal maternity care and overlooking childbearing women’s unique experiences during labour constitute D&AC. According to them, suboptimal maternity care is comprised of unconsented care, discriminatory care, and disrespecting childbearing women’s rights of confidentiality and anonymity. The midwives mostly offered practical examples to demonstrate their knowledge of D&AC. This is evident in the quotes below:

*Maybe you have two (2) clients on the ward. From their appearance, or from the type of people who come around them, you could tell one is from a wealthy family and the other from a poor background or something, and all attention is on the wealthy person. Meanwhile, the second client also needs your attention. …That is, can the patient afford treatment, care, and all attention are diverted towards that person and you look down on the person who is not able to afford much… erm, not giving the right care or the needed care. I will say that one is abuse….* [Midwife 003].

*Some people [health workers] won’t even ask for your concern when they are going to give you an injection, she won’t even ensure privacy, just turn your buttocks this way Madam, and then she injects you…There are instances where a midwife knows the name of the patient, or even if you’ve forgotten, the midwife can call the patient by the bed number, example ‘bed one’, but call the patient by their disease, example AIDS patient or TB patient lying there…Well, these can cause the patient to be so stigmatized beyond being human. Or, someone may be abjectly poor. Some people look at how a person looks like, being poor, whether the patient gets visitors or not, as a criterion when talking to them and these can lead to them being treated badly. It makes some patients feel bad and depressed.* [Midwife 004].

Further, the midwives offered their views on what constitutes a violation of patient-centred care. They stated that providers were in violation of patient-centred care when midwives act in ways toward the women who do not meet their expectation for perceived “acceptable” behaviour during labour. Examples of the views of the midwives are presented as follows:

*You would say, ‘why are you…screaming? This one [childbearing woman] is not screaming, so why are you doing that?* [Midwife 001].

*You know, someone may be a nullip, never delivered before, but can endure pain. Others cannot endure much pain. So, you can never compare that ‘Look at your sister lying there quietly, and you are here shouting your voice hoarse’.* [Midwife 008].

#### Forms of abuse—verbal, physical, and psychological

The midwives were asked to mention and explain behaviours they would generally define as D&AC. Their explanations revealed that they were aware of what constituted verbal, psychological, and physical abuse in maternity care, and some revealed that these behaviours are actually occurring at the facility. The midwives reported that insulting and shouting at childbearing women are examples of verbal abuse. Also, the midwives acknowledged that confining and ignoring childbearing women are forms of D&AC. The midwives noted that physical abuse is comprised of slapping, beating, kicking, and hitting of childbearing women. The following are the expressed views of the midwives.

*And with the verbal, that is where midwives falter a lot; when we talk, we don’t think of the impact it has on the patient, but sometimes we talk anyhow to the patient. And sometimes people, some people are more hurt with words. Some people don’t care, but some people are more hurt with words as compared to maybe the physical one.* [Midwife 002].

*I know of physical abuse, psychological, verbal, erm…Yeah. It starts with the verbal abuse whereby you are talking harshly with the patient or insulting the patient and their relatives. Yes. And with the physical, it can go to the extent of maybe hitting the patient*. [Midwife 007].

### Discriminatory care

The findings indicated that social inequalities facilitate D&AC. It emerged that the following marginalized groups were at high risk for D&AC: the non-compliant, mentally ill, HIV/AIDs+, teenagers, poor, and the general labour ward childbearing women. Childbearing women in the general labour ward were often disrespected and abused compared to their counterparts in the special ward. Special ward childbearing women pay for their services, whereas childbearing women admitted into the general labour ward most often use national health insurance to access maternity care. Regarding the neglect of or refusal to provide care to a childbearing woman who was HIV+, this midwife shared the following experience:

*It is because the mother is infected with HIV that is the reason why my colleagues didn’t want to treat her...* [Midwife 008].

Another midwife reported that a mentally ill postnatal woman was neglected by a midwife colleague:

*That one had to deal with a mentally ill patient. We had to force to clean her and fix the baby to breast…Force to clean [her because] she wouldn’t clean herself and I think she had CS done… And because she had the [mental illness] condition, like the attention wasn’t given so sort of she was rejected and now she was [left alone] there*. [Midwife 003].

Some midwives noted that poor postnatal women were often detained in a room, and they were only released after clearing their debts.

*We have a sideward like this that all the discharges who were not able to pay, whether you were a hundred or fifty [childbearing women], you will all be [detained] in this room.* [Midwife 007].

Regarding the disparity in treatments of the special ward and general ward childbearing women, this midwife had the following to say:

*You know when it comes to the special ward, most of the clients are difficult but those of us working here, you have to have patience…we exercise a lot of patience for these patients, for if you do not exercise restraint, some of them can cause trouble for you…those of us at the special ward do not encounter such problems [D&AC] because that is what we habitually do, but once one of us is transferred to the main ward and she starts exhibiting such care [respectful maternal care], the other staff will be talking behind your back, ‘it won’t take long for her to abandon her nice attitude. She is only doing this because she came from the special ward. Every turn, she says to clients, please, please, please. Every statement begins with a ‘please.’ Just wait, a nice attitude will vanish in a minute’…Yeah. The staff will be talking about you. So, if you don’t know what you are about, eventually, you will copy their attitude towards patients.* [Midwife 008].

Some midwives noted that teenage childbearing women were often mistreated compared to adults. Their experiences were reported as follows:

*Oh, (chuckling) the students were here so this one [midwife] will say something, and the other [midwife] will chip in “you, such a young girl, you are morally spoilt and got yourself pregnant. Now, [when we ask you to] lie down and let us deliver the baby, you won’t. So, what do you expect us to do to you right now?* [Midwife 011].

*Thirteen, fourteen, fifteen [years old girl], you are supposed to be in school, so what happened? And when they come and they start complaining ‘it’s painful, it’s this, it’s this’, if you had waited for a little you would have known that this is all. Didn’t you know that labour was painful, and you went to do this at this age? So, caring for an older person and the younger one, the respect that is given to the older client is different from the younger one.* [Midwife 012].

### Provider perception and blaming of childbearing women

Some midwives expect that childbearing women will come to the hospital neatly dressed and with the necessary delivery kit, be calm, lay on the bed, and comply with staff’s instructions. Also, some midwives believed that childbearing women are difficult to deal with and some intentionally act in provocative ways. From the views of the midwives, it is evident that such beliefs about childbearing women have compelled the midwives to act in unprofessional ways that disrespected and abused childbearing women.

*If it comes to the attention that you are just a petty trader in the market, to put it mildly, some of these petty traders are not exceptionally neat, not their fault but a lot are unkempt. So, when they are coming to labour, instead of taking a bath, shave, do the necessary little stuff that makes a woman presentable, she just picks a bag and presents herself to the ward. Sometimes, you open that bag and it is full of bed bugs. So, if you don’t hold yourself in check, you will get angry [and act unprofessionally]*. [Midwife 008].

Some midwives believed that the misbehaviour of childbearing women during labour was a cause for their becoming victims of D&AC. The midwives recounted that childbearing women in labour hardly follow their instructions, and this act of disrespect sometimes compel them to act out D&AC.

*The staff can sometimes look at the way someone [childbearing woman] will present herself and use that as a yardstick to respect her or not. But this can also create issues. But some of these patients are troublesome too, and that in turn cause some of the midwives to misbehave.* [Midwife 008].

### Non-evidenced based practices of preventing adverse outcome

Although these actions are non-evidence-based**,** some midwives believed that shouting, threatening, restraining, and hitting childbearing women during the active phase of labour can prevent neonatal and maternal death. This belief suggests that D&AC is internalized and normalized by these midwives.

*In the second stage when the baby is crowning and the mother is expected to give it way, due to the pain, she may not even know what she is doing and might be closing her legs up and thus hurting the baby. In such a situation, you may involuntarily hit her on the thighs and shout ‘open up!’ (Laughing at the recollections) …As for that one, we frequently do that. Sometimes it happens. It is not always the case though. Here, we have a belt that we use to strap the legs to the bedposts, so you can’t close your legs. In the absence of such devices and an expectant mother closes her legs, you can be distressed because she would be physically hurting the baby and a midwife may involuntarily hit the thighs and shout at her to open the legs wide.* [Midwife 004].

*[At the] labour ward for instance, if a person [the childbearing woman] is in the second stage, and you tend to say let me leave the patient to do whatever she wants to do until the baby comes, then you are not helping the woman and the baby as well because the baby may come out being asphyxiated. When they get to the second stage, they tend to be somehow tired, not being able to push. But if maybe you use some little force, the woman will tend to push and then you will have the baby and the mother is OK but if you leave the woman like that, she will just relax and then you may end up having an asphyxiated baby. So, in situations like that, we tend to be harsh on them for them to push.* [Midwife 008].

### Health system problems (inadequate staff, protocol, insufficient delivery beds)

The health systems related facilitators cut across human resource management, policy guidelines, and the architectural structure of the hospital. The midwives mentioned that job distress resulting from unrealistic staff-to-childbearing women ratio, lithotomy-only-birthing position guidelines, incompatibility of the hospital rooms to accommodate alternative birthing positions, and hospital policy on poor childbearing women are drivers of D&AC.

Regarding job distress, the midwives’ responses suggest that pressure from work sometimes put them in situations to act in an unprofessional manner. Some of them noted that the current staff-to-childbearing women ratio of 4 midwives to 30 childbearing women put unbearable pressure on them [midwives].

*We have on this ward, this night, thirty-three patients to four midwives, some [childbearing women] are in labour, some are eclamptic, some are having respiratory distress, and then you have the pressure, you feel the pressure, so sometimes you would react in a way which you are not supposed to, because of that pressure that is mounting on you, you might act in a weird way which you are not supposed to…sometimes, too, you would not mind the patient [ignore the childbearing woman].* [Midwife 001].

*The midwives too, we are few. Because sometimes on night duty, we have a lot of patients, and once somebody is delivering, even after the procedure itself, the documentation is another thing. And you also have to do it in as much as you have to look at the others who are in the first stage. And we are few. At most, we are four (4), four or five (5) and you can’t give the care you are supposed to give, you are tired. Not that you can’t even, but you are tired. You do a delivery, do suture, documentation, go to the next person, so we, sometimes all the four people are occupied in the four second stage beds and patients are left there alone, they are shouting; they can’t see any midwife so they will be shouting because they think we have left them alone.* [Midwife 013].

It was clear that childbearing women’s birthing position was limited to the orthodox lithotomy position. According to the midwives, childbearing women preferred the squatting birthing position. However, the midwives had countless reasons for not acquiescing to childbearing women’s preference, which included the inconvenience of assisting childbearing women’s in a squatting position, hospital protocol, and the unhygienic conditions of the floor in the ward.

*She told me the baby is coming, so I told her to lie on the floor because if she stands, the baby can hit the floor. So, I told her to lie on the floor. But this lady didn’t do it but rather, how do I do it, but rather, I don’t even know how to say it, she squatted or something and in Ghana here, or in this hospital, the patient, you are supposed to lie on your back. So, she was squatting. I told her to lie on the back. And she was like ‘no, this is what I want’. And I told her ‘you can’t do this to deliver, please, lie on your back’. So, I held her hand and I turned her to lie on her back, but this woman refused to open the thigh for me to even do the delivery.* [Midwife 010].

One midwife indicated that she delivered in a squatting position contrary to the norm. Having experienced the ease associated with squatting during delivery, she attempted advocating for it as an alternative position, but her attempt was rejected by colleagues. When she was asked whether she was satisfied with her midwifery role, she hinted that she would be satisfied if childbearing women were permitted to deliver by squatting, and she reported the labour ward was the problem because it wasn’t designed with squatting in mind. Her experiences are presented as follows:

*I am not really satisfied, especially with the birthing position. It would have been easier if patients had the option of squatting [during delivery] …the delivery couch has been shaped in a certain way that you have to lie down, on your back, and it is not easy… One time, I was talking with my colleagues about it [the squatting position], and one doctor [reproachfully] responded that ‘even delivery couch, you are not getting it, and you want to deliver in that position?’* [Midwife 002].

One midwife noted that though they wished they could provide good care to childbearing women, they were unable because of certain hospital protocols on providing care for poor childbearing women.

*In a way, we want to help…because of, let’s say, the hospital protocol and other things, maybe what you want to do to help a client, you intend to do things according to protocol. So if a client is unable to pay the bills and the hospital protocol is asking you to maybe let the person lie on the floor, put a mattress on the floor and let the person lie down, you have no option than to do what you’ve been asked to do.* [Midwife 008].

Another midwife noted that the delivery bed was occupied at the time another woman had need of it, which made it difficult for her to attend to many childbearing women in the second stage at the same time. This, she noted, prevented her from providing the needed care for one childbearing woman. This is what she said:

*I nursed a patient. When she was fully dilated and then she was calling, I was attending to someone, so I was like, ‘I am coming,’ and then when I went, the baby was out. So, I just had to assist her, cut the cord and then deliver the placenta. Then she had a tear… so, the patient said, ‘when I called you, you paid no heed, when I called you, you ignored me’…It was really hurting, [so I said] I am sorry. Here [this hospital], we have only one couch. So we manage them and we monitor them at the first stage of labour on the ward and then when they are full, we bring them here [to the couch]…We have only one delivery bed…she didn’t know because the ward extends to that far end [showing the width of the ward], so sometimes you are at the last cubicle and someone is calling from the first cubicle.* [Midwife 001].

### Everyday occurrence (prevalence)

The midwives noted that D&AC is a prevailing phenomenon at their facility. The midwives indicated that they either were a first-hand witness of colleagues acting out D&AC or they personally have been the perpetrators. Neglecting, shouting, restraining, and hitting childbearing women were forms of abuses meted out to childbearing women in labour. In some instances, midwives have interpreted a woman’s pain or distress as aggressive behaviours. It is worth mentioning that the midwives have very positive, life-saving intentions even when exhibiting these abusive behaviours. The following responses of midwives elucidate the foregoing point:

*I have done [hit] it on several occasions but when I finish and the baby come(s) out, (Laughing), [I say] Madam, I am sorry for hitting you, I wouldn’t have hit you but you would have killed your baby].* [Midwife 002].

*…I hit in-between the thighs ‘open up!’, aha, that’s the only time I hit a patient, and it is not hitting, deliberately hitting a patient…Sometimes, you would have to tie those who are aggressive, yes, you would have to tie them to the bed.* [Midwife 003].

*Oh, it happens all the time. The hitting, it is an everyday occurrence…even you [the interviewer], they [midwives] will insult you when you come here. Who are you?* [Midwife 008].

Other participants indicated that they were disrespected and abused by their fellow midwives when they were in labour at the facility.

*During my labour, the midwife insulted me, my junior.* [Midwife 002].

*Even I myself, when I went into labour, I was beaten. They hit my thighs multiple times.* [Midwife 008].

## Discussion

The study explored midwives’ descriptions and experiences of D&AC and their occurrences in professional practice. The findings indicated that the midwives were aware of D&AC, and their experiences confirm that D&AC has become part of the routine for maternity care.

Midwives’ description of disrespectful maternity care encompasses the provision of inadequate care as well as physical, psychological, and verbal forms of abuse. The midwives noted that violation of childbearing women’s rights (privacy, confidentiality, quality care, etc.), non-consented care, verbal abuse (shouting at, insulting), physical abuse (beating, slapping, kicking, restraining, and detaining), and psychological abuse (ignoring, neglecting, provision of non-person-centred care) constituted D&AC. These descriptions corroborate with existing scholarly descriptions of D&AC [17, 18, 46].

From the perspective of the midwives, childbearing women belonging to marginalized and vulnerable groups were often discriminated against during intrapartum care at the facility. The non-compliant, mentally ill, HIV/AIDs, teenage, uninformed, poor, and women admitted at the general labour wards were mistreated. For Instance, childbearing women who were unable to pay for services were detained in the facility until they cleared their bills. This finding corroborates that of a systematic review of studies traversing fourteen countries that revealed the poorest members of society who have been admitted to hospital for emergency treatment were usually detained for non-payment of hospital bills and were sometimes subjected to verbal and/or physical abuse while being detained [20]. In Ghana, detaining childbearing women or patients for non-payment of bills is against laid down guidelines governing healthcare delivery. However, the practice of detaining clients for non-payment of bills is a frequent practice in our study setting. These revelations confirmed Sen et al. (2018)‘s view that patients who belonged to marginalized and vulnerable groups in society were often at risk of D&AC in healthcare facilities [26]. Other studies also confirm the findings of our study [20–22]. These groups of women may be discriminated against because they are considered disempowered or disadvantaged because of societal perspectives. It was striking to know that a midwife from a general ward who provided D&AC to women was able to provide respectful maternal care to women when assigned to a special ward.

Midwives’ perception of childbearing women’s appearances and attitudes as well as their enforcement of non-evidenced based practices to prevent adverse childbirth outcomes expose childbearing women to DA&C. The study revealed that childbearing women were restricted from moving during labour by using stirrups to maintain them in the lithotomy position, a practice considered disrespectful and abusive [47]. The study also revealed that midwives attributed their professional misconduct during intrapartum care to the unwillingness of childbearing women to yield to their instructions. This victim blaming attitude of the midwives have been reported in another study conducted in India in which midwives blamed some of their disrespectful and abusive care practices on non-cooperative attitudes of women who visited the hospital for care [29].

Health system problems such as inadequate staff, job distress, and hospital protocol on the birthing position were identified as drivers of DA&C. Healthcare providers in other studies have given similar justifications for their engagement in D&AC on childbearing women during intrapartum care. They mentioned that inadequate clinical and support staff and weak health systems prevented them from translating their knowledge of respectful maternity care into practice [30, 31, 34, 35]. For instance, student midwives and practicing midwives in Ghana and Ethiopia reported that huge workload, burnout from job due to unrealistic staff-to-childbearing women ratio and the pressure to save mother and child during delivery can compel skilled providers to engage in practices that are deemed D&AC [30, 34]. Also, some midwives mentioned that they detained childbearing women who could not pay for services or asked them to vacate hospital beds because of internal protocols in the hospital. The patients’ charter of Ghana enjoins all healthcare providers to treat and administer care in ways that promote the dignity, welfare, and rights of the patients [48]. Also, the current Ghana midwifery curriculum covers respectful maternity care, holistic patient care, and non-conventional birthing positions with the aim of equipping student midwives on best professional practices. Despite all these, the realities of caregiving in the study setting is quite different. Anecdotal evidence indicated that hospital managers are primarily focused on good maternity outcomes, which makes the midwives to feel to use all means to ensure that both mother and baby are safe during delivery. This pressure may account for the resorting to D&AC as a means to guarantee good clinical outcomes and to avoid punitive measures in the event of neonatal and or maternal deaths. Some midwives mentioned that certain hospital protocols prevent them from discharging good care. For example, the midwives noted that many childbearing women expressed interest in other forms of birthing position apart from the lithotomy positions, but it will be difficult for midwives to allow childbearing women to assume a birth position contrary to hospital guidelines.

The study revealed that DA&C by midwives frequently occurred in the study setting. Some midwives themselves had experienced D&AC during their childbirth, and this makes them dissatisfied with the care and has subsequently informed their practice. These midwives, having had a birth experience, understood the process of labour and provided respectful care to the childbearing women. This means that midwives’ experience of childbirth may help them to appreciate childbearing women’s unique changes and experiences during labour and provide acceptable care to childbearing women.

Multiple studies that look at women’s perspective on D&AC reported that the midwives’ engagement in abuse was in their interest, but some found it dehumanizing [19, 49, 50]. For example, some studies from Nigeria mentioned that the postpartum women believed that midwives shouted, slapped, or pinched them because they wanted them to have safe delivery [49, 50]. However, a study from Ghana reported that childbearing women found disrespectful and abusive intrapartum care unacceptable regardless of the good intentions of the midwives [19].

Midwives in this study seem to lack the appropriate ways of relating to and communicating with women in labour. The current study supports a previous study by the lead author on the phenomenon, where postpartum women reported D&AC similar to those obtained in the present study. In the study, women reported they were disregarded, beaten, shouted at, and insulted by the midwives [19]. Also, postpartum women in studies in other health facilities in Ghana and elsewhere confirmed the midwives’ reports that D&AC frequently occur in healthcare facilities [21, 22, 24, 51, 52].

The results of the study have implications that are worth mentioning. Considering the complexities surrounding the hospital environment and practice, pushing for punitive measures alone as a means of ensuring respectful maternity care will achieve minimal result. Thus, we propose that the hospital should reignite her commitments to its own guidelines and protocols that are in line with the patient’s charter and other international guidelines on patient safety, autonomy and respect, and ensure that midwives comply with these directives in an effort to promote respectful intrapartum care. Secondly, childbirth settings should be resourced to allow for the use of women’s desired childbirth positions. Further, to alleviate D&AC in the study setting, the government of Ghana and other development partners would have to address the problems of understaffing and ill-equipped maternity care facilities. The D&AC can partly be dealt with through re-orientation and in-service education. Midwives would have to be thoroughly educated on respectful Patient Care including patient-centred care and be made aware of the uniqueness in the way each childbearing woman responds to pain and other physio-psychological changes during labour. We equally proposed that through media campaigns and public educations, women should be made aware of their rights and be empowered to demand for better and respectful treatment in their relationships with healthcare providers during maternity care.

The finding of our study suggests that more qualitative research is needed to understand the covert and overt facilitators of D&AC as well as quantitative labour observations in the study setting. Since the midwives in the study cited certain undocumented hospital protocols and practices that put them in positions to engage in care practices that they described as disrespectful, it will be helpful for a study to explore the views of managerial and supervisory stakeholders in the hospital on such protocols.

The authors acknowledge some limitations. Findings from an exploratory descriptive qualitative study are highly contextual. However, participants were drawn out of one institution and this may have generalizability implications. Irrespective of the above limitations an important strength of this study is that rich and in-depth information on midwives’ perspectives and experiences of D&AC in maternity care have been obtained. The findings can be used to change the maternal care practices in Ghana and West Africa because disrespectful care studies situated in other healthcare facilities in Ghana and other parts of West Africa have reported similar hospital system problems.

## Conclusions

The study explored the views of midwives on D&AC and their occurrences in professional practice. The midwives described D&AC as the provision of inadequate care and the overlooking of patient-centred care, and verbal, physical, and psychological abuse. The themes revealed that socio-economic inequalities, provider perception and victim blaming, and health system related factors facilitate D&AC.

## Supplementary information

**Additional file 1.** Interview guide.

**Additional file 2.** Codes, subthemes, and main themes.

## Data Availability

The interview transcripts used for the analyses in this study are available from the corresponding author on reasonable request.

## Abbreviations

D&AC: Disrespect and Abusive Care
LMICs: Low and Middle-Income Countries
MCH-D: Maternal and child health directorate
