# Supplementary material for: “I wouldn’t have hit you, but you would have killed your baby:” exploring midwives’ perspectives on disrespect and abusive Care in Ghana
Source: BMC Pregnancy Childbirth. 2020 Jan 6;20:15. doi: 10.1186/s12884-019-2691-y (PMC6945392; doi:10.1186/s12884-019-2691-y)
Supplement: Supplementary file 1 — Additional file 1. Interview guide. [file 12884_2019_2691_MOESM1_ESM.docx]

**Interview guide**

**Introduction**

Thank you for agreeing to share your **experiences and perception on respectful and non-abusive maternal care** with me. Before we start our conversation, I want to assure you that your interview responses will be kept confidential and will not be shared with your health care team. Your responses will be combined with that of other study participants and will not be linked with your name or any identifiable information that can be used to trace you. You may skip any questions you don’t want to answer, and you may end the interview at any time. You may also decide to withdraw from this study at any time without any consequences.

We will like to record this interview to make sure that we listen to your responses correctly. We will write down your responses verbatim or base on the meaning of your comments. We shall share the recording and the transcripts with a person who is good in Twi and English language to review your responses which we have written down. This will ensure that we have written your responses correctly. Your name will not be associated with the recording or on the transcript. We will assign you a number as your study code. If your name or any identifiable information comes on the transcript, we will delete it before sharing it with other individuals who may have access to the study data.

We will use false name to replace the number assigned to you when quoting your expressions in any part of the study or during publication of aspects of the study. We will keep all your recording and transcripts with a password and in a cabinet under lock and key for 5 years. All documents bearing any of your identifiable information will be kept separately; hence, no one can trace the documents to you. All the documents will be destroyed after the storage period. Agreeing to the interview indicates your willingness to participate in the study.

Do I have your permission to start the interview?

[If No, thank participant for time and end the session] [If yes, continue with the interview]

Thank you for consenting for participation. Before I start to record, I will ask some general questions about you.

**Biographical data**

1. Age (years):……………………………………………………….
2. Educational background:…………………………………..
3. Professional grade…………………………………………….
4. Religion:…………………………………………………………….
5. Marital status:……………………………………………………
6. Number of children:…………………………………………..
7. Years of working experience……………………………….
8. Units worked………………………………………………………

Thank you for sharing your background with me. Now, do I have your permission to start the recording?

**PUT ON YOUR RECORDER**

[If No, thank participant for time and end the session] [If yes, continue with the interview]

Now that the recording has started, please say “Yes” to confirm that you approve of me recording the interview

**Guiding questions**

1. Please, in your opinion, what is respectful care?

- Respecting the human rights of the patient
- Ethically acceptable care (autonomy, veracity, just and non-malfeasance)
- Giving patient attention during care
- Consent of patients
- Client-centered care and involving patient in care
- Non-disclosure of assessment result

1. Please, in your view, what is non-abusive care?
   - Forms of abusive care
     1. Physical
     2. Psychological
     3. Social
     4. Cognitive
     5. Economical
     6. Confinement

- Ignoring patient

1. Please, in what situation should some forms of force be applied during care?
   - Please, share some examples of such forces with me.
2. Please, have you ever encountered a difficult client in your working life?
   - Can you share your experience with me?
   - What about the client’s behavior was displeasing?
   - Please, what was your response
3. Please do you recall any situation between you and your client where the interaction was the best?
   - Can you tell me about the good about it?
   - What was the client’s perception about that best care
   - How did it improve the client’s satisfaction?
4. Please do you recall any situation between you and your client where the interaction was not the best?
   - Please, what in your view did not go well?
5. In what situation do you think patient should be respected
6. What is your view about hitting patient a little?
7. Some people say, it does not matter what goes on in labour so long as mother and baby are well at the end. What do you say?
8. Please are you satisfied with your caregiving role as a midwife?
9. Please, is there anything else you may like to share?
