# Supplementary material for: “I wouldn’t have hit you, but you would have killed your baby:” exploring midwives’ perspectives on disrespect and abusive Care in Ghana
Source: BMC Pregnancy Childbirth. 2020 Jan 6;20:15. doi: 10.1186/s12884-019-2691-y (PMC6945392; doi:10.1186/s12884-019-2691-y)
Supplement: Supplementary file 2 — Additional file 2. Codes, subthemes, and main themes. [file 12884_2019_2691_MOESM2_ESM.docx]

**Additional file 2: Codes, subthemes, and main themes**

| **Codes** | **Subthemes** | **Main themes** |
| --- | --- | --- |
| comparing patients experiences | **1.      Providing inadequate care and overlooking patient-centred care** | **Inadequate care and forms** |
| comparing patients experiences an abuse |  |  |
| aware of verbal abuse |  |  |
| confinement |  |  |
| explains why hitting patient during labor not best |  |  |
| mistreatement is abuse |  |  |
| not providing appropriate care is an abuse |  |  |
| regretful for ignoring patient during labor |  |  |
| rejecting patients during labor is an abuse |  |  |
|  |  |  |
| slapping the patient | **2.      Forms of Abuse (verbal, psychological, and physical)** |  |
| shouting at patient |  |  |
| restraining patient by holding them down |  |  |
| restraining patient by tying them |  |  |
| pinching patient during labor |  |  |
| hitting patient on the thigh |  |  |
| hitting patients |  |  |
| hitting patients during second stage of labor |  |  |
| harshly speaking to patient |  |  |
| threatening patients to calm down |  |  |
| verbally abuse difficult client |  |  |
| verbally abuse poor patients |  |  |
| verbally abused a patient |  |  |
| aware of verbal abuse |  |  |
| confinement |  |  |
|  |  |  |
| confinement of poor patients | **1.      Discriminatory care** | **Facilitators of D&AC** |
| discriminatory care |  |  |
| giving treatment base on economic status |  |  |
| giving treatment base on social status |  |  |
| midwife nice attitude associated with special ward |  |  |
| midwife tolerate special ward patients |  |  |
| special treatment for patients in special ward |  |  |
| special ward comes with charges |  |  |
| treating patients base on social rank |  |  |
| Verbally Abuse difficult clients |  |  |
| Verbally Abuse economically disadvantaged clients |  |  |
| Verbally Abuse a patient |  |  |
| Neglect hiv+ patient |  |  |
| Neglect hiv+ patient |  |  |
|  |  |  |
| experiences of good patients | **2.      Provider perception and victim blaming childbearing women/** |  |
| midwife expected patient to be remorseful |  |  |
| midwife likes patient calm during labor |  |  |
| midwives expects patients to be appreciative |  |  |
| midwife thinks teen preg women are difficult |  |  |
| dealing with difficult clients |  |  |
| dealing with difficult clients (petty traders) |  |  |
| experiences of difficult patients |  |  |
| experiences with difficult patients |  |  |
| disobedience of women cause of D&AC |  |  |
|  |  |  |
|  |  |  |
| Hitting the woman to save life | **3. Non-evidenced based practices of preventing adverse outcome** |  |
| Shouting at woman for safe delivery |  |  |
| Restraining woman for safe delivery |  |  |
|  |  |  |
| delivery ward design not compatible with squarting | **4.      Health systems related facilitators** |  |
| delivery ward unhieginic for squarting |  |  |
| long delivery ward (hall) |  |  |
| One referral point for hypertensive pregnancies |  |  |
| only one couch/delivery bed in the labor ward |  |  |
| hospital enviroment not suitable for squarting |  |  |
| few doctors on duty during weekend |  |  |
| four midwives to thirty three patients |  |  |
| normal wards assosciated with bad treatment |  |  |
| few doctors on duty during weekend |  |  |
| four midwives to thirty three patients |  |  |
| patient staff ratio reason for inadequte care |  |  |
| work pressure makes midwives react negatively |  |  |
| insulting patients is daily occurrence |  |  |
|  |  |  |
| ever witnessed colleauges abuse patients | **1.      Prevalence of D&AC** | **Prevalence of D&AC** |
| ever witnessed colleauges hit patients |  |  |
| ever witnessed colleauges verbally abuse patients |  |  |
| other staff come to beat patient for staff on duty |  |  |
| ever heard that midwives beat patients |  |  |
| ever heard that midwives cane patients in labor |  |  |
| ever hit a patient during second stage of labor |  |  |
| midwife experienced an abuse during her labor |  |  |
| midwife experienced verbal abuse during her labor |  |  |
| midwife felt embarassed when colleagues abuse her |  |  |
| hitting patient happens daily |  |  |
| ever applied force on patient |  |  |
